# Arf6 in lymphatic endothelial cells regulates lymphangiogenesis by controlling directional cell migration

**DOI:** 10.1038/s41598-017-11240-x

**Published:** 2017-09-12

**Authors:** Yueh-Chien Lin, Norihiko Ohbayashi, Tsunaki Hongu, Naohiro Katagiri, Yuji Funakoshi, Hsinyu Lee, Yasunori Kanaho

**Affiliations:** 1 Department of Physiological Chemistry, Faculty of Medicine and Graduate School of Comprehensive Human Sciences, University of Tsukuba, Tsukuba, 305-8575 Japan; 2 Department of Life Science, National Taiwan University, Taipei, Taiwan; 3 Department of Electrical Engineering, National Taiwan University, Taipei, Taiwan; 4 Institute of Biomedical Electronic and Bioinformatics, National Taiwan University, Taipei, Taiwan; 5 Center for Biotechnology, National Taiwan University, Taipei, Taiwan

## Abstract

The small GTPase Arf6 plays pivotal roles in a wide variety of cellular events such as endocytosis, exocytosis, and actin cytoskeleton reorganization. However, the physiological functions of Arf6 at the whole animal level have not yet been thoroughly understood. Here, we show that Arf6 regulates developmental and tumor lymphangiogenesis in mice. Lymphatic endothelial cell (LEC)-specific *Arf6* conditional knockout (LEC-*Arf6* cKO) mouse embryos exhibit severe skin edema and impairment in the formation of lymphatic vessel network at the mid-gestation stage. Knockdown of Arf6 in human LECs inhibits *in vitro* capillary tube formation and directed cell migration induced by vascular endothelial growth factor-C (VEGF-C) by inhibiting VEGF-C-induced internalization of β1 integrin. Finally, we found that LEC-*Arf6* cKO mice transplanted with B16 melanoma cells attenuated tumor lymphangiogenesis and progression. Collectively, these results demonstrate that Arf6 in LECs plays a crucial role in physiological and pathological lymphangiogenesis.

## Introduction

The lymphatic system plays critical roles in tissue fluid homeostasis, lipid absorption, and immune surveillance. Malfunction of this system results in a wide variety of diseases such as lymphedema, fibrosis, inflammation, and metastasis^1^. A subset of venous endothelial cells in the anterior cardinal vein of embryonic day (E) 9.5 mice start to express Prox1, the key transcription factor for differentiation of lymphatic endothelial cells (LECs)^2^. Prox1-expressing LEC progenitors in the cardinal vein of E10-11.5 mice assemble into the pre-lymphatic clusters, and migrate away from the cardinal vein wall to form lymph sacs and superficial lymphatic vessels^2–5^. The sprouting from the lymph sac is induced by vascular endothelial growth factor-C (VEGF-C) through its receptor VEGFR3^6^, which regulates receptor modulators such as Neuropilin 2^7^, Ephrin B2^8^ and β1 integrin^9–11^ to generate the lymphatic vascular network in embryos within E14.5. From E15.5 to the postnatal stage, the primary lymphatic vascular networks undergo remodeling to form the mature lymphatic network composed of initial lymphatic vessels, pre-collectors and collecting lymphatic vessels^12^. Although several guidance molecules, cellular interactions, and extrinsic forces have been reported to be important for developmental lymphangiogenesis^13^, the molecular mechanisms for lymphatic vascular network formation are poorly understood.

The small GTPase Arf6 plays pivotal roles in a wide variety of cellular events, such as endocytosis, exocytosis, and actin cytoskeleton reorganization^14, 15^. Arf6 cycles between the GDP-bound inactive and GTP-bound active forms. This cycle is precisely regulated by guanine nucleotide exchange factors (GEFs) and GTPase-activating proteins (GAPs)^16^. Arf6 exists as the inactive form at the resting state of cells, and is activated by Arf6-specific GEFs, which promote the exchange of GDP with GTP on Arf6, in response to agonist stimulation to interact with its downstream effector molecules and regulate their activities and subcellular localization, thereby transducing signals downstream. The activated Arf6 consequently regulates membrane trafficking, including endocytosis and recycling of receptors and cell adhesion molecules, and membrane ruffle formation through actin cytoskeletal reorganization^14, 15^. Thereafter, GTP on Arf6 is hydrolyzed to GDP by GTPase activity of Arf6 stimulated by Arf6-specific GAPs. Thus, cellular functions of Arf6 have been well documented. However, the physiological functions of Arf6 at the whole animal level had not yet been well elucidated. To address this issue, we have generated *Arf6* knockout (*Arf6* ^−/−^) mice that are embryonic lethal at midgestation with liver development defect^17^. To further investigate the physiological functions of Arf6 *in vivo*, we have generated tissue- and cell type-specific *Arf6* conditional knockout mice, and demonstrated that Arf6 in vascular endothelial cells and neurons in the central nervous system plays critical roles in HGF-induced tumor angiogenesis^18^ and neuronal myelination^19^, respectively.

Here we re-examined the different stages of *Arf6* ^−/−^ embryos in depth, and confirmed that *Arf6* ^−/−^ embryos exhibit severe edema in their head and back. Since the edema is closely related to the defect in lymphatic vascular networks, we generated tamoxifen-inducible LEC-specific *Arf6* conditional knockout (LEC-*Arf6* cKO) mice, and investigated physiological functions of Arf6 in LECs. Similar to *Arf6* ^−/−^ embryos, these mouse embryos showed the edema on their back with the defective lymphatic network. Moreover, we found that Arf6 regulates VEGF-C-dependent directional cell migration, adhesion, and *in vitro* capillary tube formation through regulating the internalization of surface β1 integrin. Finally, we found that lymphangiogenesis in the tumor produced by B16 melanoma cells and tumor progression are inhibited in the tamoxifen-inducible LEC-*Arf6*-cKO mice. Thus, we clarified for the first time the novel physiological and pathological functions of Arf6 in LECs *in vivo*, providing the opportunity for a potential clinical application to anti-cancer treatment.

## Results

### Ablation of *Arf6* from lymphatic endothelial cells causes edema and defect in lymphatic vascular network formation

**Figure 1:**
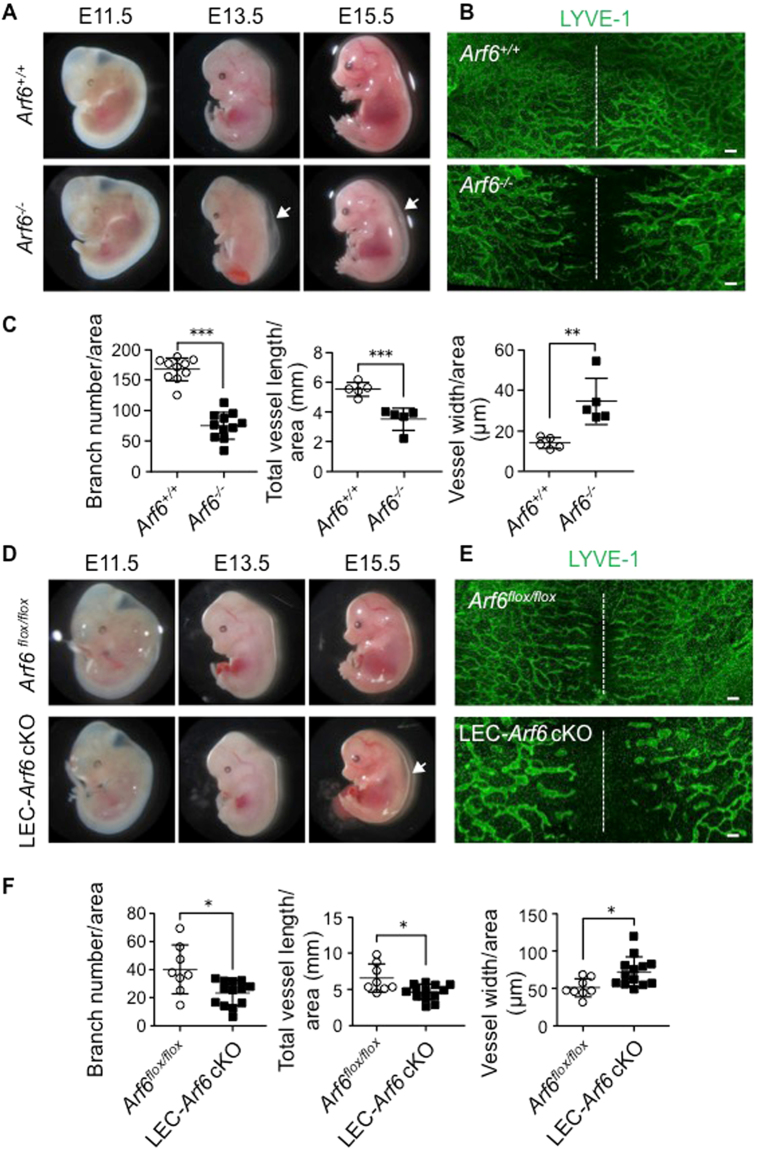
*Arf6* ^−/−^ and LEC-*Arf6* cKO mice induce dorsal skin edema and abnormal lymphatic vascular network. **(A**,**D)** Appearance of *Arf6* ^−/−^ and LEC-*Arf6* cKO embryos in comparison with that of control *Arf6* ^*+/+*^ and *Arf6* ^*flox/flox*^ embryos, respectively. Note that the edema (white arrows) on the back of E13.5 and 15.5 *Arf6* ^−/−^ embryos and of E15.5 LEC-*Arf6* cKO was induced. **(B**,**E)** Aberration of dorsal subcutaneous lymphatic vascular network in E15.5 *Arf6* ^−/−^ and LEC-*Arf6* cKO embryos. Lymphatic vessels were immunostained for LYVE-1 (green). White dashed lines indicate the dorsal midline of the embryo. **(C**,**F)** Immunostained images of lymphatic vessels shown in **(A)** were quantified for branch number of lymphatic vessels/area (left panel), lymphatic vessel length/area (middle panel), and width of lymphatic vessels/area (right panel) in control *Arf6* ^*+/+*^ and *Arf6* ^−/−^ embryos **(C)** and control *Arf6* ^*flox/flox*^ and LEC-*Arf6* cKO embryos **(F)**. Area of 2250 × 1700 μm on both sides of the midline was measured. Each point represents individual value: n = 10 for both embryos in the left panel and n = 5 for both embryos in the middle and right panels of **(C)**, and n = 8 for *Arf6* ^*flox/flox*^ embryos and n = 13 for LEC-*Arf6* cKO embryos in (**F**). Statistical significance was assessed using student’s *t*-test. *P < 0.05, **P < 0.01, ***P < 0.005. Scale bar, 200 μm.

As we have previously reported^18^, re-examination of *Arf6* ^−/−^ embryos confirmed that knockout of *Arf6* induces the dorsal skin edema in E13.5 and E15.5 embryos (Fig. 1A). Because impairment in lymphatic vascular formation causes hydrops fetalis with back skin edema^20–23^, we examined the lymphatic vascular network formation in the back skin of *Arf6* ^−/−^ embryos by immunofluorescent staining for the specific marker of mouse LECs (mLECs) LYVE-1 (Fig. 1B). As expected, *Arf6* ^−/−^ embryos showed the aberrant morphology of the lymphatic vascular network: extension of the front tip of lymphatic vessels toward the dorsal midline was delayed in *Arf6* ^−/−^ embryos. Detailed analysis revealed the fewer branch points, shorter total vessel length, and enlarged lymphatic vessels in *Arf6* ^−/−^ embryos (Fig. 1C). In support of these observations, embryonic Prox1^+^ and LYVE-1^+^ mLECs isolated from dorsal skins of E16.5 embryos (Supplementary Fig. 1A) expressed the Arf6 protein and its mRNA (Supplementary Fig. 1B,C). In addition, Arf6 was expressed in the lymph sac of E13.5 embryos (Supplementary Fig. 1D).

The defect of lymphangiogenesis observed in *Arf6* ^−/−^ embryos and the expression of Arf6 in mLECs led us to hypothesize that Arf6 in mLECs functions in development lymphangiogenesis. To test this hypothesis, we generated tamoxifen-inducible LEC-*Arf6* cKO mice. Arf6 would be successfully deleted from mLECs in LEC-*Arf6* cKO mice by tamoxifen treatment, since the tdsRed signal was detected in the lymphatic vessels of *R26GRR;Prox1-CreER;Arf6* ^*flox/+*^ mice treated with tamoxifen (Supplementary Fig. 2). Consistent with the results obtained with *Arf6* ^−/−^ mice, E15.5 tamoxifen-treated LEC-*Arf6* cKO mice showed edema, delay of the lymphatic vessel extension, and defects in the branch points, vessel length and vessel width (Fig. 1D–F). The phenotypes observed in *Arf6* ^−/−^ and tamoxifen-treated LEC-*Arf6* cKO embryos were not due to the defect in blood vessel formation as was shown in Supplementary Fig. 3. Although heart development disorder is known to cause embryonic edema, tamoxifen-treated LEC-*Arf6* cKO embryos did not show any heart defect in the histological analysis (Supplementary Fig. 4). Taken together, these results strongly suggest that Arf6 in mLECs is essential for the developmental lymphangiogenesis.

### Arf6 plays a role in the formation of lymph sacs

**Figure 2:**
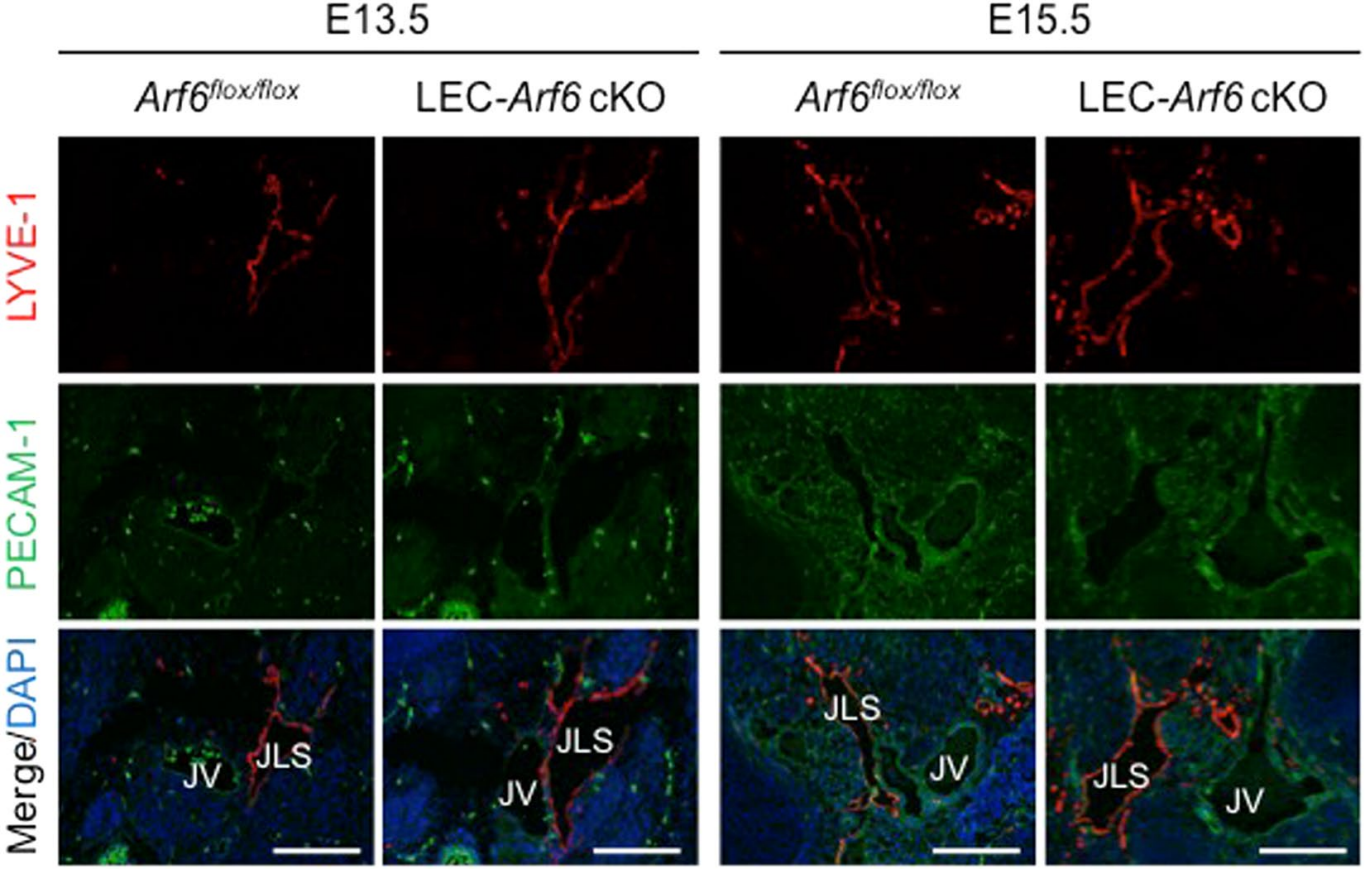
Jugular lymph sacs enlarge in LEC-*Arf6* cKO mice. Transverse jugular sections of E13.5 and E15.5 control *Arf6* ^*flox/flox*^ and LEC-*Arf6*-cKO embryos were immunostained for LYVE-1 (red), PECAM-1 (green) and DAPI (blue). Note that the LYVE-1 positive jugular lymph sac (JLS) in LEC-*Arf6* cKO embryos enlarged compared with that in control embryos. JV, jugular vein. JLS, jugular lymph sac. Scale bar, 200 μm.

To examine the functions of Arf6 in an early event of developmental lymphangiogenesis, we analyzed primary lymph sac formation. In tamoxifen-treated E13.5 and E15.5 LEC-*Arf6* cKO embryos, LYVE-1^+^ and PECAM-1^+^ lymph sacs were enlarged compared with those in control *Arf6* ^*flox/flox*^ embryos (Fig. 2).

**Figure 3:**
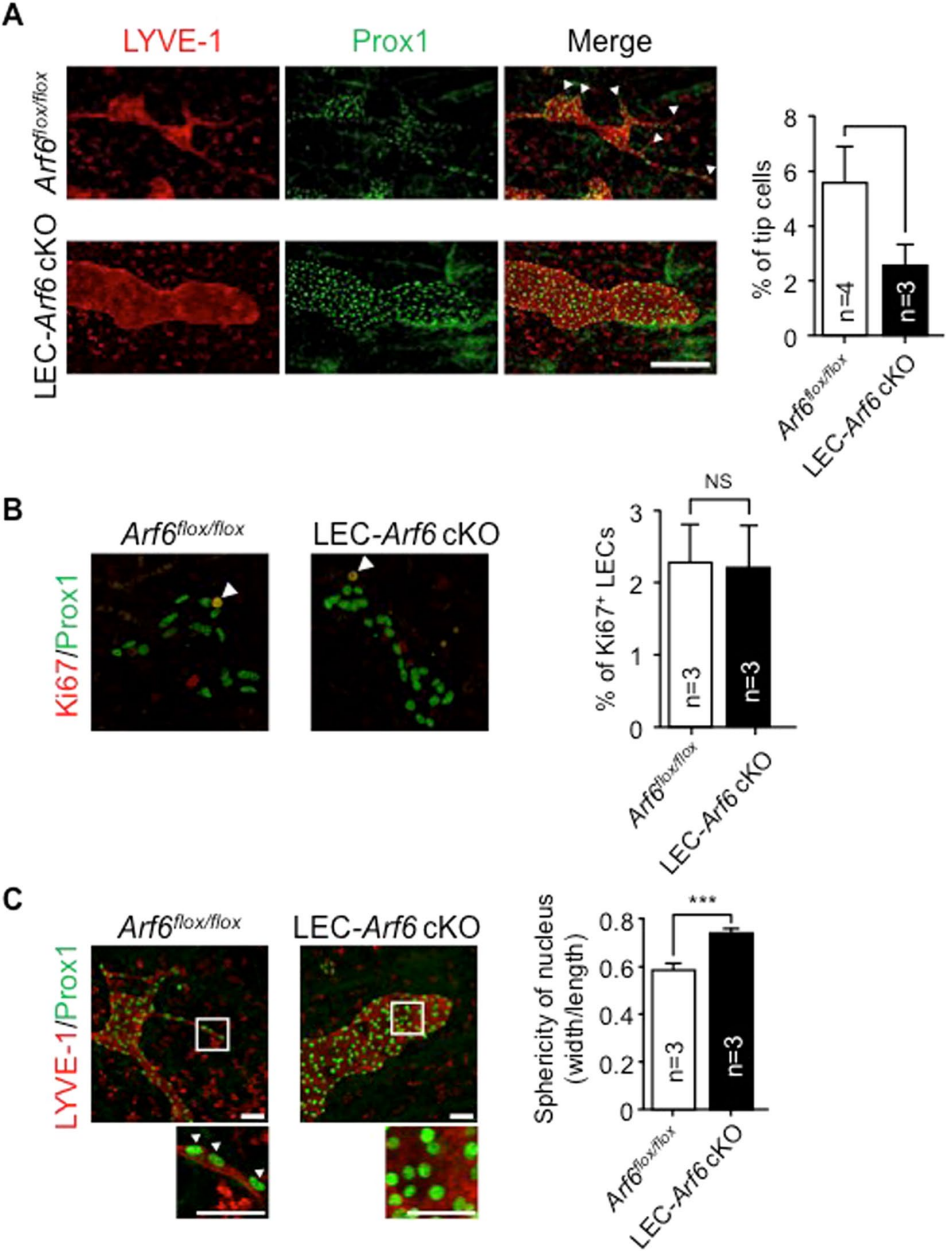
Arf6 regulates lymphatic vessel sprouting and tip cell morphology. **(A)** Representative images of sprouting lymphatic vessels in E15.5 control *Arf6* ^*flox/flox*^ (n = 4) and LEC-*Arf6* cKO (n = 3) (left panels). Transverse jugular sections were co-immunostained for LYVE-1 (red) and Prox1 (green). Arrowheads indicate sprouts of lymphatic vessels. Immunostained images shown in the left panels were quantified for the number of Prox1^+^ lymphatic tip cells in the distal migration front area of lymphatic vessels (right panel). Scale bar, 200 μm. **(B)** Representative images of proliferating mLECs in the subcutaneous lymphatic vessel of E15.5 control *Arf6* ^*flox/flox*^ (n = 5) and LEC-*Arf6* cKO (n = 5) embryos co-immunostained for Prox1 (green) and Ki67 (red) (left panels). Arrowheads represent Prox1^+^/Ki67^+^ proliferating mLECs (left panel). Percentages of Prox1^+^/Ki67^+^ proliferative mLECs of total Prox1^+^ mLECs in the distal migrating front area of lymphatic vessels were quantified (right panel). **(C)** Representative images of subcutaneous lymphatic vessels in E15.5 control *Arf6* ^*flox/flox*^ (n = 3) and LEC-*Arf6*-cKO embryos (n = 3) co-immunostained for LYVE-1 (red) and Prox1 (green). Lower panels are magnified images of the square area in the upper panels. Arrowheads in the magnified images indicate the oval nucleus. Immunostained images were quantified for sphericity of nucleus (width/length) (right panel). Statistical significance was assessed using student’s *t*-test. NS, not significant, *P < 0.05, ***P < 0.005. Scale bar, 200 μm **(A**,**B)** and 25 μm **(C)**.

Sprouting of the lymphatic vessel from lymph sacs is the second step in the developmental lymphangiogenesis to form the lymphatic vascular network. Arf6 appeared to play a function in the sprouting of lymphatic vessels, since tamoxifen-treated LEC-*Arf6* cKO embryos lacked any sprouting tips and showed enlarged LYVE-1^+^ and Prox1^+^ lymphatic vessels (Fig. 3A). The enlargement of lymphatic vessels was not due to the enhancement of mLEC proliferation as assessed by immunostaining of developing lymphatic vessels for the proliferation marker Ki67 (Fig. 3B). This result was supported by the finding that knockdown of Arf6 in hLECs was without effects on hLEC proliferation *in vitro* (Supplementary Fig. 5). Moreover, it was found that the nuclei of mLECs in tamoxifen-treated LEC-*Arf6* cKO embryos were spherical, while those of control mLECs were oval (Fig. 3C), suggesting that Arf6 regulates sprouting by controlling the cell migration. These results demonstrate that Arf6 in mLECs plays an important role in sprouting from the lymph sac to form the lymphatic vascular network.

### Arf6 in mLECs promotes *in vitro* capillary tube formation by regulating cell migration upon VEGF-C stimulation

**Figure 4:**
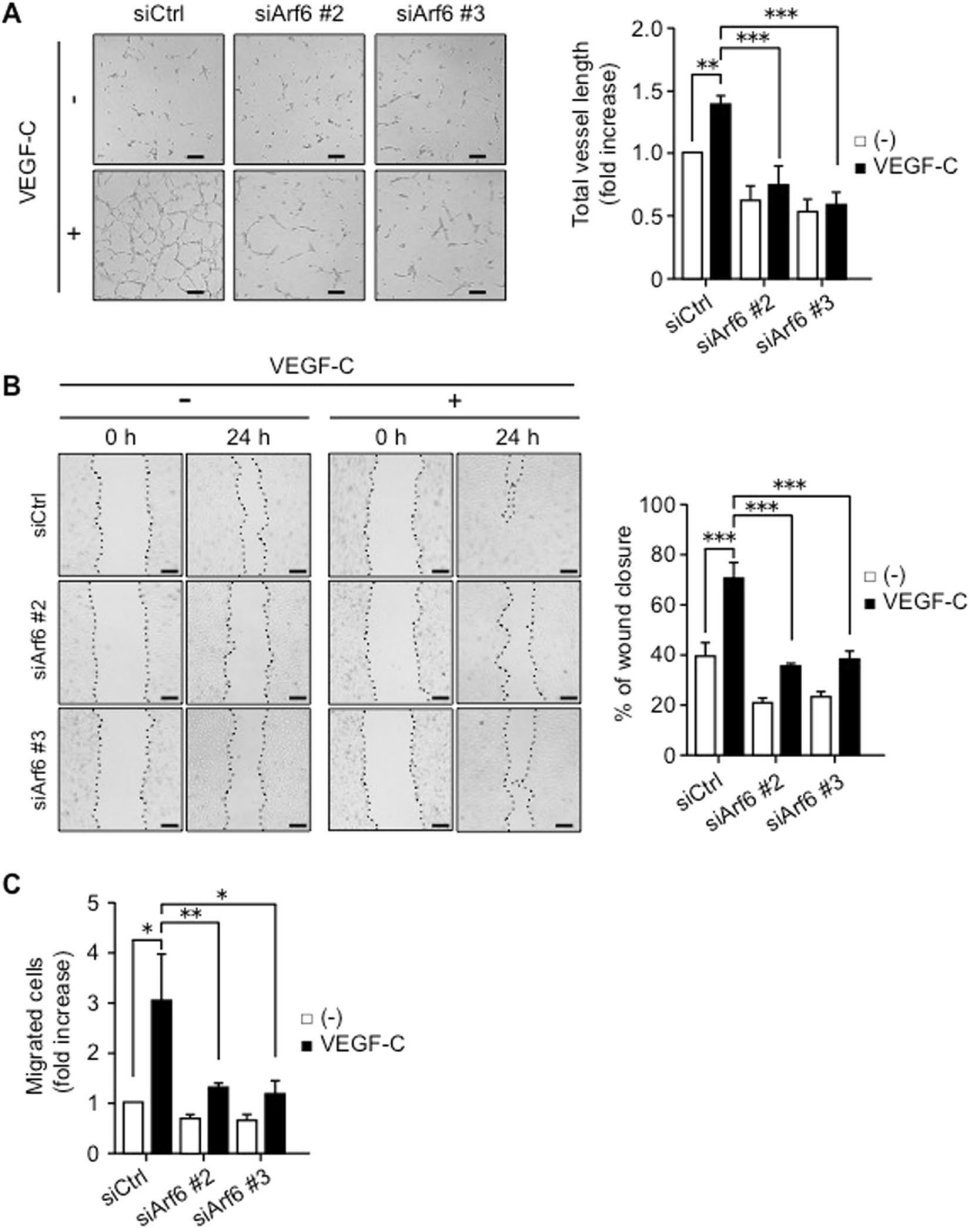
Arf6 regulates *in vitro* capillary tube formation and cell migration of hLECs. **(A)** Representative images of *in vitro* capillary tube formation by control and Arf6-knocked-down hLECs (left panels), and quantified data for total tube length (right panel). hLECs were transfected with siRNAs for 48 hours and starved overnight. The control and Arf6-knocked-down hLECs were stimulated without or with 200 ng/ml of VEGF-C for 24 hours. Total vessel length was calculated in 4 fields/experiments of three independent experiments. **(B)** Representative images of wound healing by control and Arf6-knocked-down hLECs (left panels), and quantified data for percentages of wound closure (right panel). Confluent monolayers of hLECs transfected with control and *Arf6* siRNAs were scratched and immediately treated without or with 200 ng/ml of VEGF-C, and images were obtained at 0 and 24 hours after wounding. Dotted lines in the images indicate the border of the wound. Wound closure was measured in 4 fields/experiments of four independent experiments and represented as percentages of wound distance during 24 hours. **(C)** Cell migration of control and *Arf6*-knocked-down hLECs stimulated without or with 200 ng/ml of VEGF-C. Cells migrated to the lower surface of the membrane filter in transwell migration chamber were stained with DAPI. DAPI-positive cells in four fields per sample were counted, and the data were shown as means ± SEM from at least 3 independent experiments. Statistical significance was assessed using one-way ANOVA. *P < 0.05, **P < 0.01, ***P < 0.005. Scale bar, 200 μm.

It has been reported that VEGF-C signaling regulates lymphatic vascular development^24^. To address a question whether Arf6 regulates VEGF-C-dependent lymphatic vascular formation, we investigated the involvement of Arf6 on the VEGF-C-dependent *in vitro* capillary tube formation by hLECs. As was expected, knockdown of Arf6 in hLECs (Supplementary Fig. 5A) impaired the VEGF-C-induced tube formation (Fig. 4A).

Since VEGF-C signaling regulates migration of LECs^24, 25^, which is an essential cell event for lymphatic vascular development, we examined whether Arf6 regulates hLEC migration by wound healing and transwell migration assays. At 24 hours after wounding, the VEGF-C-dependent wound closure was significantly delayed in Arf6-knocked-down hLECs (~40% closure) compared with control (~75% closure) (Fig. 4B). Transwell migration of hLECs stimulated by VEGF-C, which was used as a chemoattractant, was also markedly inhibited by knockdown of Arf6 (Fig. 4C). Thus, Arf6 appeared to play an important role in the cell migration.

### Arf6 regulates directional cell migration of LECs

**Figure 5:**
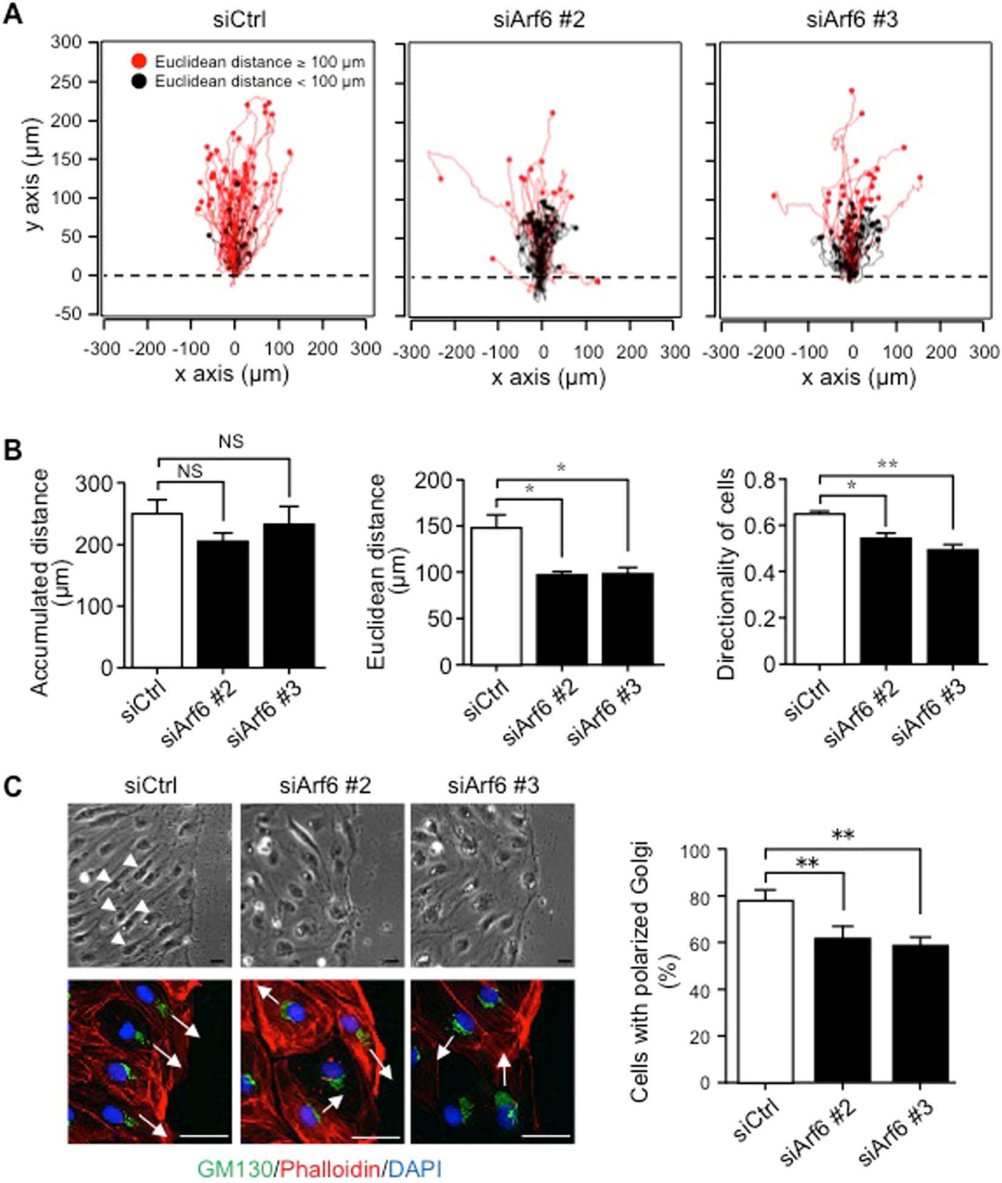
Arf6 knockdown causes defect in directed cell migration of hLECs. **(A)** Tracking of cell migration during wound healing by time-lapse video microscopy. hLECs transfected with siRNAs for 48 hours were grown to the confluence, scratched, and stimulated with 200 ng/ml of VEGF-C. Images were obtained every 10 minutes for 20 hours after wounding. Each line represents the trajectory of an individual cell. Red and black lines indicate the euclidean distance over and less than 100 μm, respectively. Dotted lines indicate the boarder of the wound. Each experiment was performed four times, and at least 60 cells per experiment were analyzed. **(B)** Images shown in **(A)** were quantified for accumulated distance (left panel), euclidean distance (middle panel), and directionality (right panel), which was calculated by dividing euclidean distance by accumulated distance. **(C)** Representative phase contrast microscopic images of control and Arf6-knocked-down of hLECs (upper images) and immunostained images with anti-GM130 antibody (green), phalloidin (red), and DAPI (blue) (lower images) at the wounded edge (left panels). Arrowheads indicate elongated cells. Arrows indicate the migrating direction of the cell as decided by the location of Golgi. Images were quantified for cells with polarized Golgi (right panel). At least 200 cells per experiment were analyzed, and data were shown as means ± SEM from at least 3 independent experiments. Statistical significance was assessed using one-way ANOVA. NS, not significant, *P < 0.05, **P < 0.01. Scale bar, 25 μm.

To further investigate whether Arf6 regulates the directional cell migration, time-lapse tracking of hLEC movement in response to VEGF-C stimulation was analyzed (Fig. 5A). Although knockdown of Arf6 did not affect accumulated distance, euclidean distance and directionality of cells were significantly reduced by Arf6 knockdown (Fig. 5B). Thus, Arf6 is required for the directional cell migration but not for unsophisticated cell movement.

Interference with the directed cell migration by Arf6 knockdown may be resulted from the disturbance of cell polarity, since Golgi orientation, which has been shown to be involved in the cell polarity^22, 23, 26^, was randomized in Arf6-knocked-down hLECs (Fig. 5C); although migrating control cells at the wounded site elongated (left top panel, arrow heads), Arf6-knocked-down cells remained spherical (middle and right top panels), which is consistent with the results shown in Fig. 3C that the nuclei of mLECs in tamoxifen-treated LEC-*Arf6* cKO embryos showed spherical shape while those of control mLECs was oval. Taken together, these results suggest that Arf6 in LECs regulates directional cell migration by controlling cell polarity.

### Arf6 regulates internalization of β1 integrin in LECs

**Figure 6:**
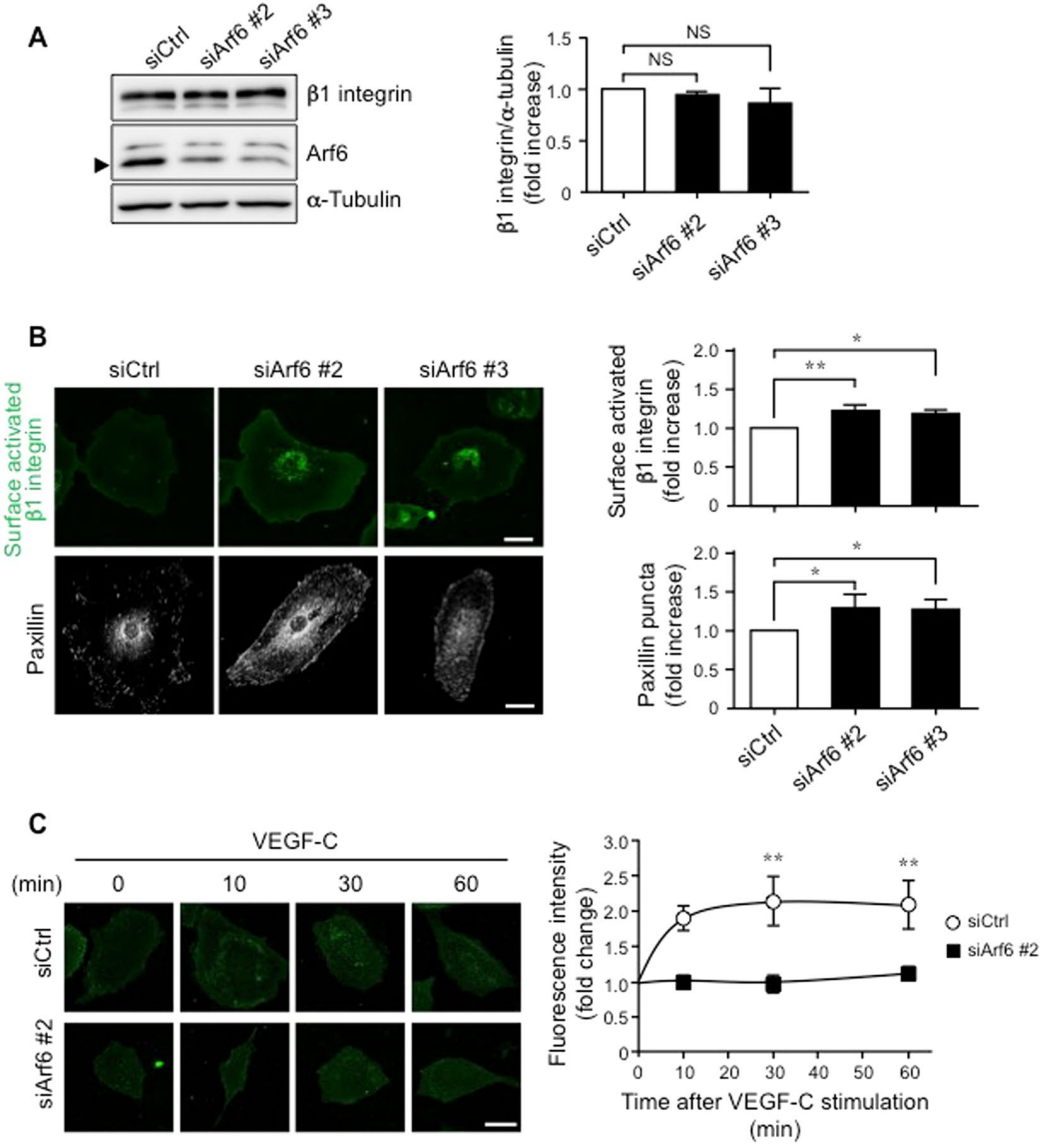
Arf6 regulates β1 integrin internalization in hLECs. hLECs were transfected with siRNAs for 48 hours and subjected to the experiments. **(A)** Western blots of β1 integrin levels in control and Arf6-knocked-down hLECs (left panel), and their quantified data (right panel). An arrowhead indicates the position of endogenous Arf6. **(B)** Representative images of surface activated β1 integrin (green, upper panels) and paxillin (white, lower panels) in control and Arf6-knocked-down hLECs (left panels), and quantified data for their levels (right panels). **(C)** Representative images of time-dependent β1 integrin internalization (left panels) and its quantified data (right panel). The internalized β1 integrin was shown in green. Data were shown as means ± SEM from at least 3 independent experiments. Statistical significance was assessed using One-way ANOVA. NS, not significant, *P < 0.05, **P < 0.01. Scale bar, 25 μm **(B)** and 50 μm **(C)**. The full-length blotting images of (**A**) are presented in Supplementary Fig. 7.

Impairment of the internalization of cell surface β1 integrin in nascent endothelium disrupts arterial endothelial cell polarity and lumen formation^27–29^. In addition, it has been reported that Arf6 signaling regulates the internalization of β1 integrin^30, 31^. These reports led us to examine whether Arf6 is involved in the β1 integrin internalization in LECs. The total amount of β1 integrin in hLECs was not affected by knockdown of Arf6 (Fig. 6A). When the levels of active form of surface β1 integrin and the adhesion molecule paxillin were analyzed, levels of both molecules were significantly increased in Arf6-knocked-down hLECs (Fig. 6B). In addition, β1 integrin internalization promoted by VEGF-C stimulation of hLECs in a time-dependent manner was almost completely inhibited by knockdown of Arf6 (Fig. 6C). These results, taken together, suggest that Arf6 regulates VEGF-C-induced cell polarity and directional cell migration by controlling β1 integrin internalization in LECs.

### Ablation of *Arf6* from LECs interferes with tumor lymphangiogenesis and cancer progression

**Figure 7:**
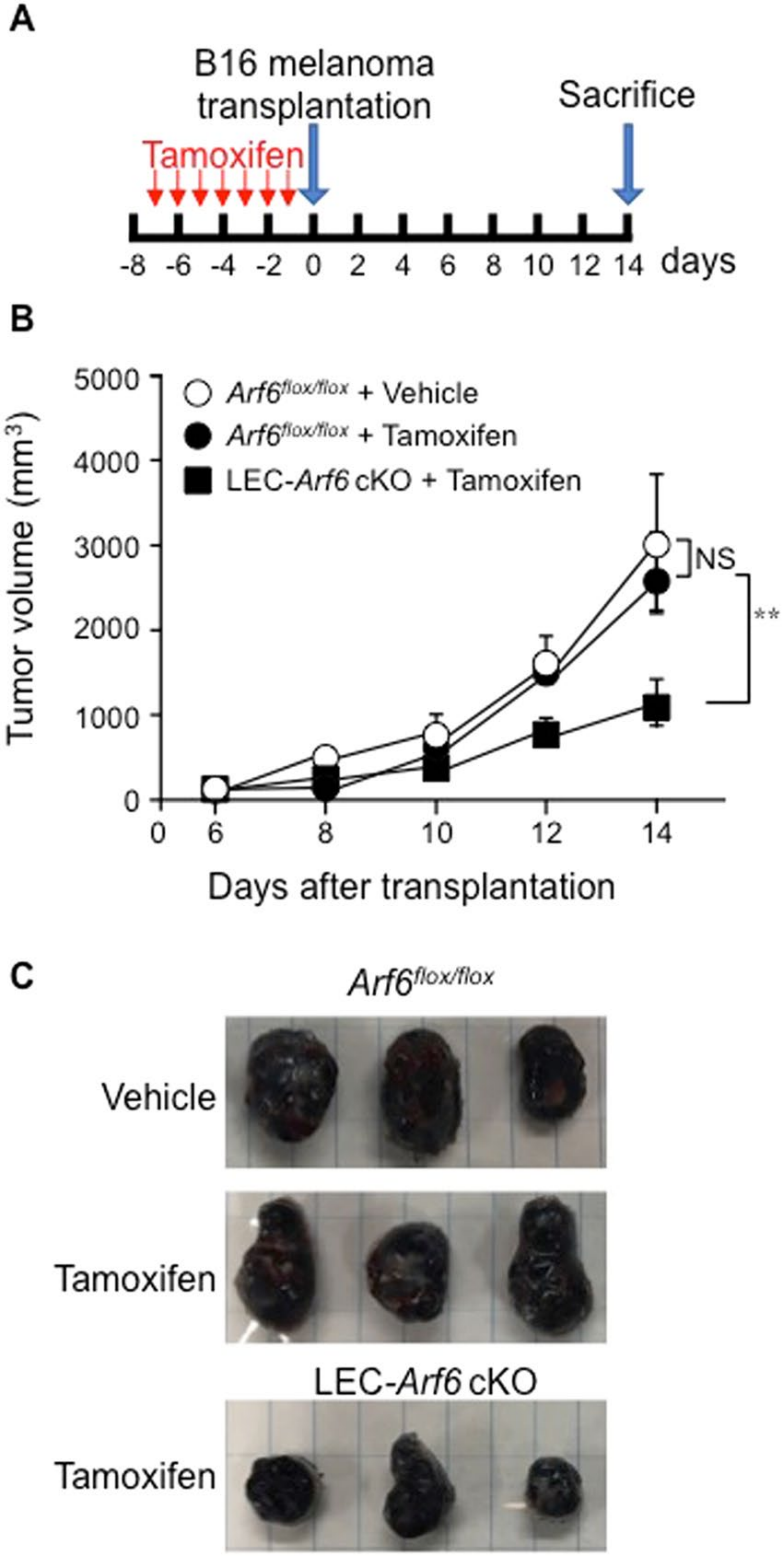
The effect of Arf6 ablation from LECs on tumor progression. **(A)** Scheme of the assay for the effect of Arf6 ablation from mLECs on tumor progression. Tamoxifen (3 mg) was daily injected into the peritoneal cavity of the mice for one week, and B16 melanoma cells (2 × 10^4^ cells) were subcutaneously transplanted into the right lower back region of the mice. The size of the tumor was measured by digital caliper every two days from day 6 after the transplantation. **(B)** Tumor volumes produced in vehicle-treated *Arf6* ^*flox/flox*^, tamoxifen-treated *Arf6* ^*flox/flox*^, and tamoxifen-treated LEC-*Arf6* cKO mice were measured according to the schedule described in **(A)**. **(C)** At 14 days of the transplantation, tumors were dissected, and 3 examples were shown. The data shown in **(B)** are mean ± SEM from three independent experiments. Statistical significance was assessed using One-way ANOVA. NS, not significant. **P < 0.01.

Cancer progression and lymphatic metastasis are tightly related with tumor lymphangiogenesis^32, 33^. These reports and the results shown above raised a possibility that Arf6 in LECs is involved in tumor lymphangiogenesis and cancer progression. To address these issues, B16 melanoma cells were transplanted into tamoxifen-treated control and LEC-*Arf6* cKO mice (Fig. 7A). Tamoxifen was without effect on tumor growth, since tumor growth in *Arf6* ^*flox/flox*^ mice administered with tamoxifen was almost the same as that in these mice administrated with vehicle (Fig. 7B,C). The tumor volume produced was significantly reduced by knockout of *Arf6* in mLECs (Fig. 7B,C). Correlated to this result, tumor lymphangiogenesis was suppressed by *Arf6* knockout (Supplementary Fig. 6). Thus, Arf6 in mLECs plays an important role in tumor lymphangiogenesis, thereby regulating cancer progression. These results provide a new cancer therapeutic opportunity.

## Discussion

The results obtained in this study demonstrate for the first time that the small G protein Arf6 in LECs plays an important role in the VEGF-C-induced directional cell migration, which is critical for lymphatic vascular network formation, by regulating β1 integrin internalization, providing insight into the molecular mechanism of developmental lymphangiogenesis. Furthermore, we clarified that Arf6 in LECs plays pivotal roles in tumor lymphangiogenesis and cancer progression, giving a new cancer therapeutic opportunity. Thus, our findings in this study provide insight into not only physiological but also pathological functions of Arf6 in LECs.

Although Arf6 has been well established to regulate the integrin recycling^30, 34, 35^, the molecular mechanisms for VEGF-C-dependent, Arf6-mediated β1 integrin recycling in LECs remained unclear. Activation of Arf6 in LECs in response to VEGF-C stimulation could be essential for the integrin recycling. This assumption is supported by the report that the Arf6-specific GEF GEP100 promotes the β1 integrin endocytosis to enhance the cell attachment to and spreading on the β1 integrin substrate fibronectin^30^. Recently, we have reported that Arf6 is activated by the GTPase dynamin2, which promotes scission of the invaginated plasma membrane in a manner dependent on its conformational change induced upon GTP hydrolysis^36^. The Arf6 activation by dynamin2 is mediated by the Arf6-specific GEFs such as EFA6A, EFA6B, and EFA6D during clathrin-mediated endocytosis^36^. If it is the case that these Arf6 GEFs are involved in VEGF-C-stimulated Arf6 activation in LECs, dynamin2 mediates VEGF-C-dependent Arf6 activation through an Arf6 GEF(s). This issue remains to be clarified.

What is the cellular signaling downstream of the activated Arf6 coupling to the β1 integrin endocytosis in VEGF-C-stimulated LECs? It is plausible that the lipid kinase phosphatidylinositol 4-phosphate 5-kinase (PIP5K), which is directly activated by the active form of Arf6 to produce the versatile signaling lipid phosphatidylinositol 4,5-bisphosphate (PI4,5P_2_) at the plasma membrane^14^, is involved in this cellular signaling as an effector molecule of Arf6 activated by VEGF-C stimulation. The PIP5K product PI4,5P_2_ regulates activities of actin-binding proteins such as the actin severing and capping protein gelsolin, thereby reorganizing actin cytoskeleton to facilitate endocytosis of membrane proteins^37–39^. Alternatively, phospholipase D1 (PLD1) which produces the signaling lipid phosphatidic acid (PA) may function as a mediator for the active form of Arf6 coupling to β1 integrin endocytosis. This idea is derived from the reports that PLD1 is directly activated by Arf6 in response to agonist stimulation^40, 41^, and its product PA facilitates endocytosis by forming the membrane curvature at the neck of the deeply invaginated membrane^42, 43^. In addition to these possible functions of PIP5K and PLD1 in β1 integrin endocytosis, these two lipid-metabolizing enzymes mutually accelerate their activation by a positive feedback mechanism: PA is necessary for the activation of PIP5K by Arf6^43^, and PI4,5P_2_ supports PLD1 activation by Arf6^44^.

Besides Arf6, other proteins may be involved in VEGF-C-stimulated β1 integrin endocytosis to regulate directional cell migration. For example, the endocytic adaptor protein Numb has been reported to bind β1 integrin and control integrin endocytosis for directional cell migration with aPKC and PAR-3^45^. Thus, the molecular mechanism of β1 integrin endocytosis seems to be very complicated, and it is of interest to clarify the precise molecular mechanism for VEGF-C-dependent β1 integrin endocytosis, especially crosstalk of cellular signaling pathways in this cell event.

The edema on the back of *Arf6* ^−/−^ mice was observed at E13.5 (Fig. 1A), while LEC-*Arf6* cKO embryos showed the edema at E15.5 but not at E13.5 (Fig. 1D). This difference in the embryonic days inducing edema between these two types of *Arf6*-deficient mice would be attributable to the involvement of another type(s) of cells distinct from LECs in an earlier step of lymphangiogenesis. This idea is supported by the report that the lymphatic vascular system predominantly originates from the vein at earlier stage of development (E7.5) before lymphatic vessels are formed at E9.5 in mouse embryos^46^. Moreover, it has been reported that non-venous mesenchymal cells also contribute to an earlier step (s) of lymphatic development as a source of LECs^47^. These observations explain well why we did not observe any defects in the lymphatic vessel formation in the VEC-specific *Arf6* cKO mice (data not shown), which had been generated previously^18^, and why the edema was induced in *Arf6* ^−/−^ embryos earlier than in LEC-*Arf6* cKO embryos: ablation of *Arf6* from both venous and non-venous mesenchymal cells in *Arf6* ^−/−^ embryos might induce the lymphedema earlier than LEC-*Arf6*-cKO.

Finally, we demonstrated in this study that Arf6 in LECs is a key molecule for tumor lymphangiogenesis and cancer progression (Fig. 7 and Supplementary Fig. 6). Tumor lymphangiogenesis, as well as tumor angiogenesis, is a potential therapeutic target for cancer treatment^40, 41^. We have recently reported that Arf6 expressed in VECs plays an important role in tumor angiogenesis and cancer progression: ablation of *Arf6* from mouse vascular endothelial cells (mVECs) inhibits tumor angiogenesis, therefore suppressing tumor growth^18^. Thus, Arf6 in pan-endothelial cells is a highly potential therapeutic target to prevent cancer progression. However, recent preclinical studies have suggested that anti-angiogenic therapy promotes cancer metastasis by inducing hypoxia in cancer cells^48, 49^. It is noteworthy that Arf6 expressed in breast cancer cells is required for cancer metastasis^50^. Thus, Arf6 plays critical roles in tumor lymphangiogenesis/angiogenesis and cancer metastasis. These reports and our results obtained in this study suggest that an inhibitor(s) of Arf6 could efficiently prevent both tumor progression and metastasis. Specific inhibitors of Arf6 might provide a new cancer therapeutic opportunity.

## Materials and Methods

### Mice

*Arf6* ^−/−^ and *Arf6* ^*flox/flox*^ mice were generated as described previously^17–19^. Tamoxifen-inducible LEC-*Arf6* cKO mice were generated by mating *Arf6* ^*flox/flox*^ and *Prox1-CreER* ^*T2*^ mice, which were kindly provided by Dr. S. Ito (Showa Pharmaceutical University, Japan)^3^. To initiate Cre-mediated recombination in embryos, pregnant mice were intraperitoneally injected with 3 mg of tamoxifen dissolved in sunflower oil every day from E10.5^3^. *ROSA26-CAGp-loxP-EGFP-loxP-tdsRed* (*R26GRR*) mice provided by Dr. S. Takahashi (University of Tsukuba, Japan) were used to validate the Cre activity in LECs^51, 52^. All experiments using mice were performed according to the Guidelines for Proper Conduct of Animal Experiments, Science Council of Japan, and the protocols were approved by the Animal Care and Use Committee of University of Tsukuba.

### Whole-mount immunofluorescence staining of embryonic dorsal skin

After mouse embryos were fixed with 4% paraformaldehyde (PFA)/PBS at 4 °C and subsequently dehydrated in methanol, the dorsal skin were dissected, rehydrated in PBST (0.2% Tween-20/PBS), and incubated in the blocking buffer consisting of 0.1 M Tris-HCl, pH7.5, 0.5% blocking reagent (PerkinElmer Life Sciences) and 0.15 M NaCl for 1 hour at room temperature (r.t.). The skin samples were incubated with the primary antibodies against LYVE-1 (Abcam), Prox1 (R&D Systems), PECAM-1 (BD Biosciences), or Ki67 (Abcam) at 4 °C overnight and subsequently with Alexa Fluor^®^-488-, Alexa Fluor^®^-546-, Alexa Fluor^®^-594-, Cy3-, and Cy5-conjugated secondary antibodies (Thermo Fisher Scientific). Immunofluorescence images were obtained with Biozero BZ-X700 microscope (Keyence, Japan) or Leica TCS SP5 confocal microscope. The branch number, total vessel length, and width of lymphatic vessels were analyzed by NIH ImageJ software.

### Tissue sections and immunohistochemical analysis

Embryos were fixed with 4% PFA/PBS at 4 °C overnight, equilibrated in 30% sucrose/PBS, then embedded in OCT compound. Embedded embryos were cryosectioned at 14–16 μm, and subjected to immunohistochemical analysis. For the immunohistochemical analysis, sections were washed with PBS, and blocked with the blocking buffer at r.t. for 1 hour. Sections were then incubated with anti-Arf6, which was kindly provided by Dr. H. Sakagami (Kitasato University, Japan), anti-LYVE-1, anti-Prox1, or anti-PECAM-1 antibody at 4 °C overnight. After washing with PBST, sections were incubated with Alexa Fluor^®^-488 goat anti-rabbit IgG antibody at r.t. for 1 hour, and counterstained with DAPI. Images were obtained with Biozero BZ-X700 microscope.

### Cell culture and siRNA transfection

Human LECs (hLECs; Lonza) were cultured in EGM^TM^-2 MV Medium (Lonza), and maintained within 5 passages. siRNAs for Arf6 (Dharmacon) were transfected with Lipofectamine 2000 (Thermo Fisher Scientific) for 48 hours according to the manufacturer’s instruction. B16 melanoma cells were maintained in Dulbecco’s modified Eagle medium (DMEM; Nacalai Tesque, Japan) supplemented with 10% fetal bovine serum, penicillin and streptomycin.

### *In vitro* capillary tube formation by hLECs

hLECs transfected with siRNAs for Arf6 for 48 hours were starved overnight on 24-well plates coated with the growth factor-reduced Matrigel (BD Biosciences) at 1.5 × 10^5^ cells per well, and stimulated with 200 ng/ml of VEGF-C (R&D Systems). After 24 hours of the stimulation, cell images were obtained using the Biozero BZ-X700 microscope, and the capillary tube length was measured by NIH ImageJ software.

### Assays for wound healing and cell migration

For the wound healing assay, hLECs transfected with siRNA for Arf6 were grown on 24-well plates to full confluency and starved in serum-free EBM^TM^-2 Basal Medium (Lonza) overnight. Monolayers of the cells were scratched with pipette tips, and stimulated with 200 ng/ml of VEGF-C. Cell images were obtained with the Biozero BZ-X700 microscope and wound closure was analyzed by NIH ImageJ software.

For the transwell migration assay, hLECs starved as described above were seeded in the upper chamber of transwell migration chamber (8 μm pore size; Corning) at 2 × 10^4^ cells per chamber. The lower chamber was filled with 200 ng/ml of VEGF-C/EBM^TM^-2 Basal Medium. At 12 hours after seeding, membrane filters were fixed with 4% PFA/PBS and stained with DAPI. hLECs migrated to the lower surface of the membrane filter were imaged using the Biozero BZ-X700 microscope, and cell number was counted.

For time-lapse analysis of wound-induced cell migration, hLECs transfected with siRNA for Arf6 were cultured on the μ-Dish dish (ibidi, Germany) to full confluency, and starved in serum-free EBM^TM^-2 Basal Medium overnight. The monolayer of the hLECs was scratched with pipette tips, and then incubated in the humidified chamber of a time-lapse microscopy (FLUOVIEW FV10i, Olympus) in the presence or absence of 200 ng/ml of VEGF-C at 5% CO_2_ and 37 °C. Cell migration at the wounded area was recorded every 10 minutes for 20 hours by tracking the nucleus using the manual-tracking tool of NIH ImageJ. Cell trajectories were analyzed using the Chemotaxis and Migration Tool Software (ibidi). Accumulated distance was calculated as the sum of all cell movement. Euclidean distance represents the straight distance between the start and end point of cell migration. Directionality was calculated by dividing euclidean distance by accumulated distance.

### Western Blotting

Western blotting was carried out as previously reported^18^, using anti-Arf6 that was previously generated by us^53^, anti-β1 integrin TS2/16 (Santa Cruz) and anti-α-tubulin (Sigma-Aldrich) antibodies.

### Assay for the activated β1 integrin level and focal adhesion formation

To evaluate the activated β1 integrin levels at the plasma membrane, control and Arf6-knocked-down hLECs were stimulated with VEGF-C. Cells were fixed with 4% PFA/PBS, blocked with the blocking buffer, then immunostained by sequential incubation with anti-active β1 integrin (9EG7, BD Biosciences) and Alexa Fluor^®^-488-conjugated secondary antibodies (Thermo Fisher Scientific). Immunofluorescence images were obtained with Biozero BZ-X700 microscope, and the fluorescence intensities for the activated β1 integrin were analyzed by NIH ImageJ software.

For focal adhesion (FA) formation assay, control and Arf6-knocked-down hLECs were stimulated with VEGF-C for the indicated times. Cells were fixed, blocked, and stained with anti-paxillin (BD Biosciences) and Alexa Fluor^®^-488-conjugated secondary antibodies. Images were obtained by Leica TCS SP5 confocal microscope, and the FA formation was analyzed by ImageJ software.

### Internalization of β1 integrin

The internalization of β1 integrin was performed according to previous report with minor modification^18^. Briefly, control and Arf6-knocked-down hLECs were incubated with anti-β1 integrin TS2/16 antibody for 30 minutes on ice. The β1 integrin/antibody complex on the plasma membrane was allowed to be internalized by incubating with 200 ng/ml of VEGF-C at 37 °C for the indicated time in the presence of 0.6 μM of primaquine, an inhibitor for the recycling of β1 integrin to the plasma membrane. Anti-β1 integrin antibody on the cell surface was removed by washing with the ice-cold stripping solution (0.5% acetic acid, 0.5 M NaCl and 0.05% BSA). The cells were fixed with 4% PFA/PBS, permeabilized with 0.1% Triton X-100/PBS, and visualized for the internalized β1 integrin with Fluor^®^-488-labeled secondary antibody. Z stack fluorescence images were obtained with Leica TCS SP5 confocal microscope, and the fluorescence intensities of internalized β1 integrin were analyzed by NIH ImageJ software.

### Tumor lymphangiogenesis and tumor progression

LEC-*Arf6* cKO mice were administered with 3 mg of tamoxifen into peritoneal cavity once a day for 7 days, and subcutaneously transplanted with B16 melanoma cells (2 × 10^6^ cells) suspended in 100 ml of serum-free DMEM into the dorsal flank. From day 6 after the transplantation, tumor volumes were measured by digital caliper and calculated using the following formula: tumor volume = length × width^2^ × 0.52. After 14 days of the transplantation, tumors were dissected, fixed with 4% PFA/PBS and subjected to assay for tumor lymphangiogenesis.

### Statistical analysis

Student’s *t*-test and ANOVA were used to calculate the statistical significance between two experimental groups and more than two experimental groups, respectively. The value of *P* < 0.05 was considered as statistical significance. Each result was obtained from at least three independent experiments, and all quantitated data are represented as mean ± SEM.

## Electronic supplementary material

Supplementary Information
