# Supplementary material for: Arf6 in lymphatic endothelial cells regulates lymphangiogenesis by controlling directional cell migration
Source: Sci Rep. 2017 Sep 12;7:11431. doi: 10.1038/s41598-017-11240-x (PMC5595869; doi:10.1038/s41598-017-11240-x)
Supplement: Supplementary file 1 — Supplementary Information [file 41598_2017_11240_MOESM1_ESM.pdf]

Supplemental Material

Arf6 in lymphatic endothelial cells regulates lymphangiogenesis by controling directional cell migration

Yueh-Chien Lin[1,2], Norihiko Ohbayashi[1], Tsunaki Hongu[1], Naohiro Katagiri[1], Yuji Funakoshi[1], Hsinyu Lee[2-5], Yasunori Kanaho[1,*]

[1]Department of Physiological Chemistry, Faculty of Medicine and Graduate School of Comprehensive Human Sciences, University of Tsukuba, Tsukuba 305-8575, Japan. [2]Department of Life Science, [3]Department of Electrical Engineering, [4]Institute of Biomedical Electronic and Bioinformatics and [5]Center for Biotechnology, National Taiwan University, Taipei, Taiwan

*Correspondence: Dr. Yasunori Kanaho, Department of Physiological Chemistry, Faculty of Medicine and Graduate School of Comprehensive Human Sciences, University of Tsukuba, 1-1-1 Tennodai, Tsukuba 305-8575, Japan.

Tel.: +81-29-853-3282; FAX: +81-29-853-3271; E-mail: ykanaho@md.tsukuba.ac.jp

Supplemental Materials and Methods

Isolation of primary mLECs and mVECs: Skins dissected from E16.5 embryos were digested with the solution consisting or 1 mg/ml of deoxyribonuclease I, 2.5 mg/ml of collagenase type II and type IV, 20% FBS, and 10 mM HEPES buffer in DMEM. Macrophages and hematopoietic cells were removed by incubating the cell suspension with rat anti-F4/80 (MCA497GA, Bio-Rad) and -CD45 antibody (B122583, BioLegend) and subsequently precipitating with goat anti-rat IgG microbeads (Miltenyi Biotec). mLECs and mVECs in the cell suspension were captured by rabbit anti-LYVE-1 (ab14917, Abcam,) and rat anti-PECAM-1 antibody (102501, BioLegend), and precipitated with goat anti-rabbit IgG microbeads (Miltenyi Biotec). Captured mLECs and mVECs were sorted using The MiniMACS™ kit (Miltenyi Biotec) as described previously.[56] Isolated mLECs and mVECs were immediately used for detection of mRNAs and proteins.

Reverse transcription and quantitative real-time PCR: Total cellular RNA was extracted from the cells using the TRIzol® Reagent (Life Technologies, Carlsbad, CA). The reverse transcription of the RNA (1.5 μg) was performed in a 30 μl reaction mixture using SuperScriptTM III Reverse Transcriptase (Thermo Fisher Scientific) and oligo-dT primers. Quantitative real-time PCR (qRT-PCR) reactions were conducted in Applied Biosystems 7500/7500 Fast Real-Time PCR System (Thermo Fisher Scientific) using THUNDERBIRD™ SYBR® qPCR Mix (TOYOBO) according to manufactory's instruction. The thermal profile of PCR was at 95°C for 3 min, followed by 40 cycles at 95°C for 30 sec and at 60°C for 30 sec. The sequences of paired primers for qRT-PCR are as follows: *Actin beta (Actb)* forward 5'-GATCATTGCTCCTCCTGAGC-3', *Actb* reverse 5'-GTCATAGTCCGCCTAGAAGCAT-3'; *Prox1* forward 5'-CTGGGCCAATTATCACCAGT-3', *Prox1* reverse 5'-GCCATCTTCAAAAGCTCGTC-3'; *Lyve1* forward 5'-TGGTGTTACTCCTCGCCTCT-3', *Lyve1* reverse 5'-TTCTGCGCTGACTCTACCTG-3'; *Flt1* forward 5'-AGCACCTTGACCTTGGACAC-3', *Flt1* reverse 5'-CAGGGGATGATGAGCTGTCT-3'; *Arf6* forward 5'-TGCCTAAACTGGAGGAAACTTGAA-3', *Arf6* reverse 5'-ACCACATCTCACCTGCAACATT-3'; *GAPDH* forward 5'-AAGGTGAAGGTCGGAGTC-3', *GAPDH* reverse 5'-TGTAGTTGAGGTCAATGAAGG-3'.

Cell proliferation assay: For the cell proliferation assay, siRNA-transfected hLECs were cultured in EGM^{TM}-2 MV Medium on 35-mm dishes for two days and harvested by Accutase treatment (Nacalai Tesque), and the cell number was counted.

Supplemental Figure 1

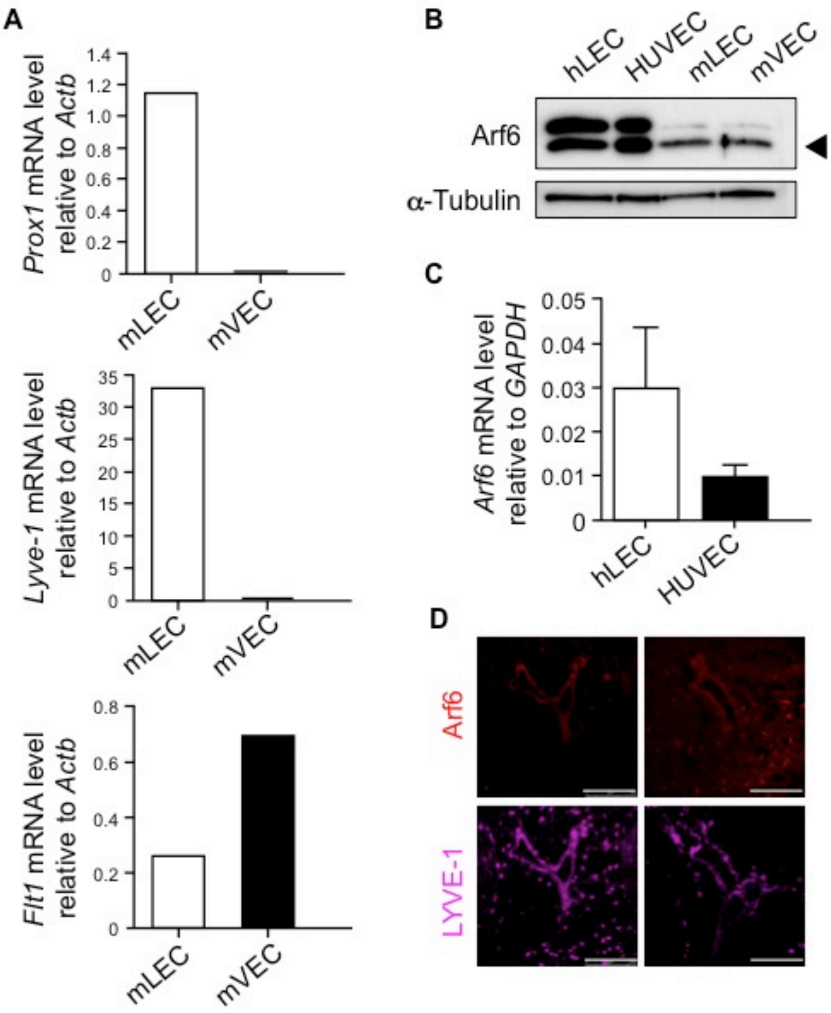

Supplemental Figure 1. Arf6 is expressed in mLECs. (A) Verification of mLECs and mVECs purified from E16.5 embryos by qRT-PCR for the lymphatic markers Prox1 and Lyve-1, and the blood vessel marker Flt1. (B) The expression levels of Arf6 protein in hLECs, HUVECs, mLECs, and mVECs determined by Western blotting. (C) *Arf6* mRNA expression levels in hLECs and HUVECs analysed by qRT-PCR. The data were shown as mean ± SEM from three independent experiments. Statistical significance was assessed using unpaired Student's *t*-test. **P* < .05. (D) Expression of Arf6 (red) in LYVE-1-positive transverse jugular lymph sacs (purple) of E13.5 mouse embryos. Scale bars, 200 μm.

Supplemental Figure 2

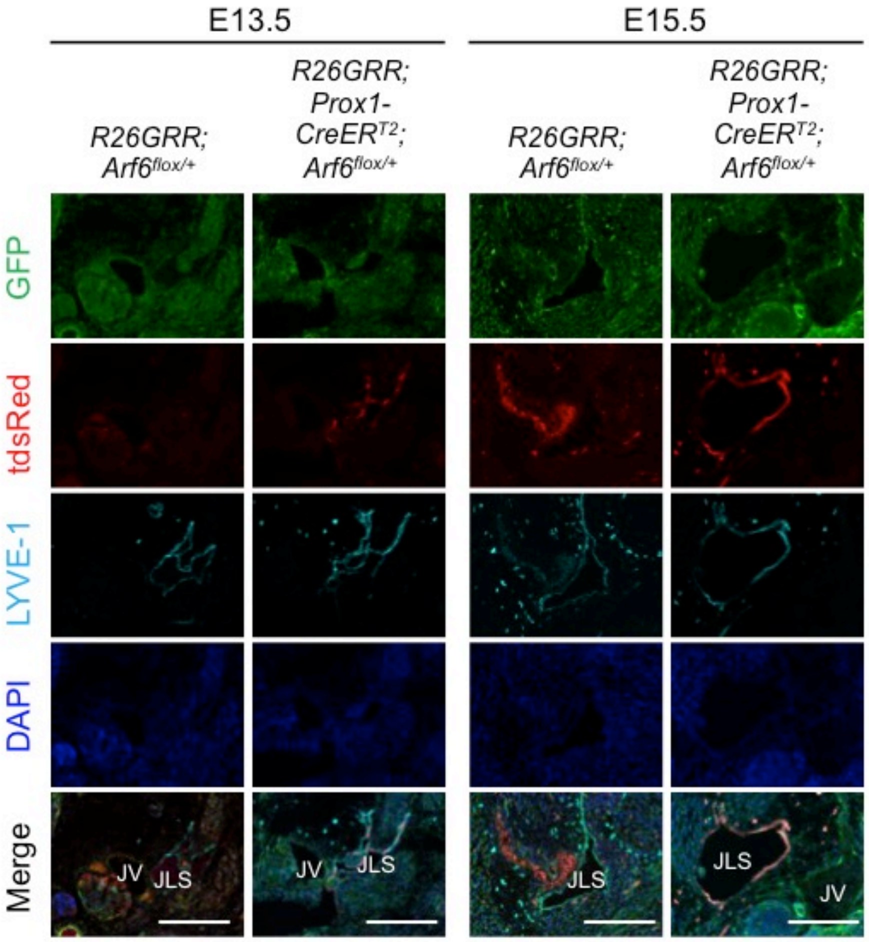

Supplemental Figure 2. Cre recombinase expression in mLECs of the jugular lymph sac. Transverse jugular sections prepared from E13.5 and E15.5 GFP-expressing control *R26GRR;Arf6*$^{flox/+}$ and *R26GRR;Prox1-CreER*^{T2}*;Arf6*$^{flox/+}$ embryos, which were administrated with tamoxifen, were stained for LYVE-1 (cyan) and DAPI (blue). tdsRed signal (red) indicates the tamoxifen-stimulated Cre recombinase activity. JLS, jugular lymph sac. IJV, internal jugular vein. Scale bars, 200 μm.

Supplemental Figure 3

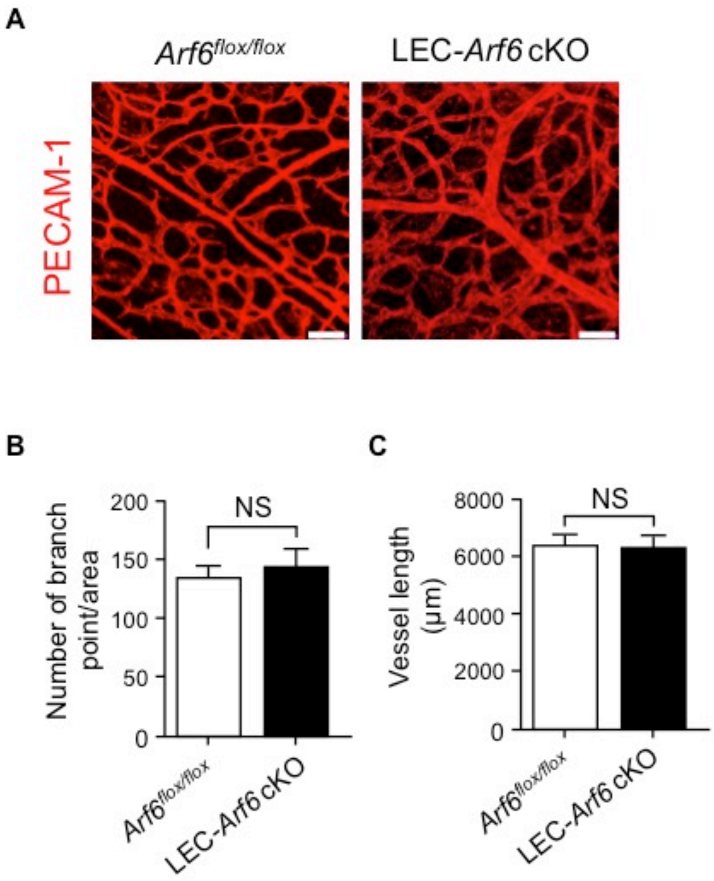

Supplemental Figure 3. mLEC-specific *Arf6* knockout does not impair the blood vessel formation. (A) Confocal z-stack images of dorsal view of subcutaneous blood vessels stained for PECAM-1 in E15.5 control *Arf6*$^{flox/flox}$ and LEC-*Arf6*-cKO embryos. (B) Quantification of branch points of blood vessels/area (393 × 393 μm) in control *Arf6*$^{flox/flox}$ (n = 3) and LEC-*Arf6*-cKO (n = 3) embryos. (C) Quantification of total blood vessel length/area (393 × 393 μm) in control *Arf6*$^{flox/flox}$ (n = 3) and LEC-*Arf6*-cKO (n = 3) embryos. The data shown were the mean ± SEM from at least three independent experiments. Statistical significance was assessed using Student's *t*-test. NS, not significant. Scale bar, 50 μm.

Supplemental Figure 4

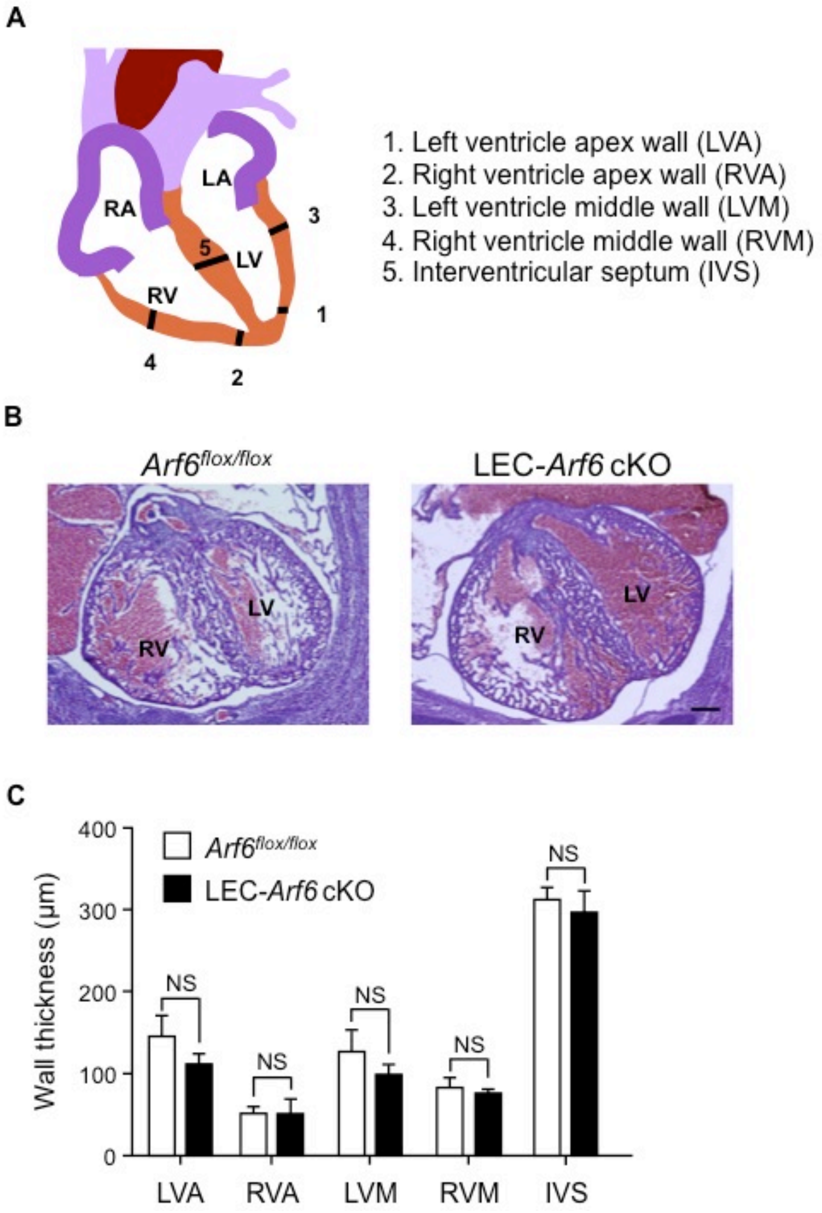

Supplemental Figure 4. LEC-*Arf6* cKO mice do not show heart failure. (A) Schematic anatomy of the heart in E15.5 embryos. LA, left atrium. LV, left ventricle. RA, right atrium. RV, right ventricle. (B) Hematoxylin & Eosin staining of transverse heart sections of E15.5 control *Arf6*$^{flox/flox}$ and LEC-*Arf6*-cKO embryos. (C) Quantification of the thickness of left ventricle apex wall (LVA), right ventricle apex wall (RVA), left ventricle middle wall (LVM), right ventricle middle wall (RVM) and interventricular septum (IVS) in E15.5 control *Arf6*$^{flox/flox}$ and LEC-*Arf6*-cKO embryos. The data shown are mean ± SEM from at least three independent experiments. Statistical significance was assessed using Student's *t*-test. NS, not significant. Scale bar, 200 μm.

Supplemental Figure 5

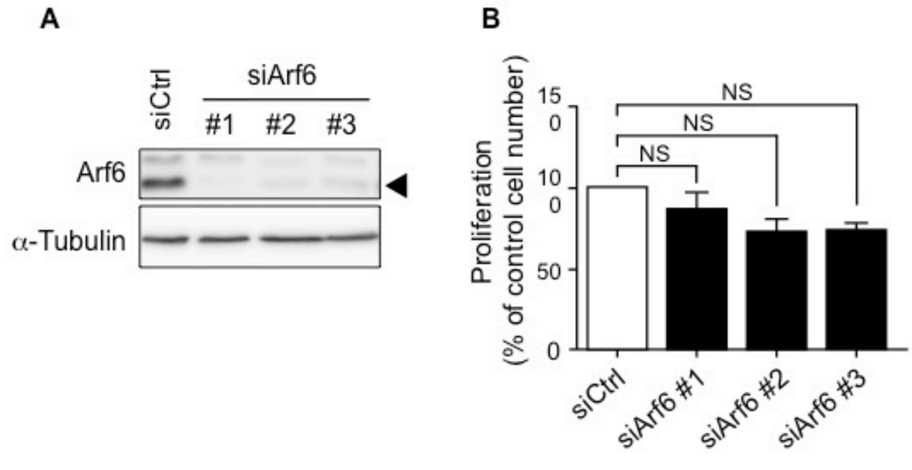

Supplemental Figure 5. Arf6 is not involved in cell proliferation of hLECs. (A) Knockdown of Arf6 in hLECs. (B) Effect of Arf6 knockdown on the cell proliferation of hLECs. After two days of cell culture, cell number was counted. The data shown are mean ± SEM from five independent experiments. Statistical significance was assessed using One-way ANOVA. NS, not significant.

Supplemental Figure 6

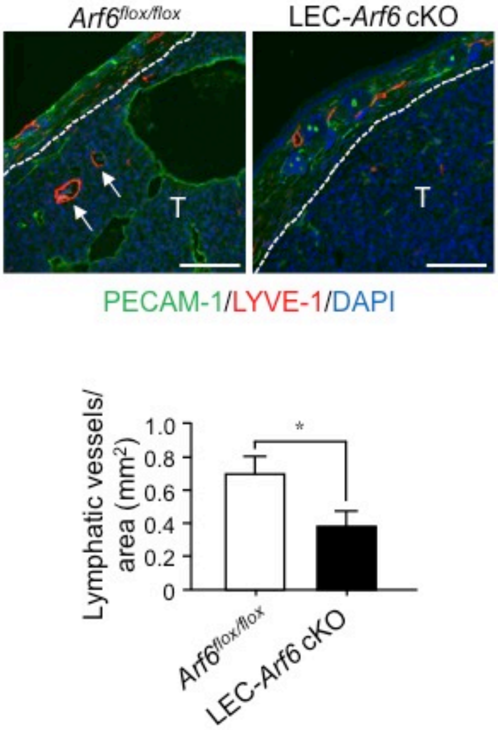

Supplemental Figure 6. Arf6 in mLECs positively regulates tumor lymphangiogenesis. Representative images of lymphatic vessels in B16 melanoma tumors produced in control *Arf6*$^{flox/flox}$ (n = 3) and LEC-*Arf6* cKO mice (n = 3). Tumor sections were co-immunostained for LYVE-1 (red) and PECAM1 (green) (upper panels). Arrows and the letter T indicate the lymphatic vessel and the tumor, respectively. White dashed lines are the border between the tumor and dermis. Quantification of percentages of lymphatic vessels/area (1525 × 1140 μm area) (lower panel) was calculated in 10 fields of images/experiments. Data shown were the mean ± SEM from at least 3 independent experiments. Statistical significance was assessed using student's *t*-test, *P < .05. Scale bar, 200 μm.
